# A Comparison of Epithelial Cells, Fibroblasts, and Osteoblasts in Dental Implant Titanium Topographies

**DOI:** 10.1155/2012/687291

**Published:** 2012-01-12

**Authors:** Fu-Yuan Teng, Chia-Ling Ko, Hsien-Nan Kuo, Jin-Jia Hu, Jia-Horng Lin, Ching-Wen Lou, Chun-Cheng Hung, Yin-Lai Wang, Cheng-Yi Cheng, Wen-Cheng Chen

**Affiliations:** ^1^Department of Dentistry, Kaohsiung Armed Forces General Hospital, Kaohsiung 802, Taiwan; ^2^School of Dentistry, College of Dental Medicine, Kaohsiung Medical University, Kaohsiung 807, Taiwan; ^3^Medical Device Development Division, Metal Industries Research & Development Centre, Kaohsiung 82151, Taiwan; ^4^Department of Biomedical Engineering, National Cheng Kung University, Tainan 701, Taiwan; ^5^Laboratory of Fiber Application and Manufacturing, Department of Fiber and Composite Materials, Feng Chia University, School of Chinese Medicine, China Medical University, Taichung 40724, Taiwan; ^6^Institute of Biomedical Engineering and Material Science, Central Taiwan University of Science and Technology, Taichung 40601, Taiwan; ^7^Department of Fiber and Composite Materials, College of Engineering, Feng Chia University, 100 Wenhwa Road., Seatwen, Taichung 40724, Taiwan

## Abstract

The major challenge for dental implants is achieving optimal esthetic appearance and a concept to fulfill this criterion is evaluated. The key to an esthetically pleasing appearance lies in the properly manage the soft tissue profile around dental implants. A novel implant restoration technique on the surface was proposed as a way to augment both soft- and hard-tissue profiles at potential implant sites. Different levels of roughness can be attained by sandblasting and acid etching, and a tetracalcium phosphate was used to supply the ions. In particular, the early stage attaching and repopulating abilities of bone cell osteoblasts (MC3T3-E1), fibroblasts (NIH 3T3), and epithelial cells (XB-2) were evaluated. The results showed that XB-2 cell adhesive qualities of a smooth surface were better than those of the roughened surfaces, the proliferative properties were reversed. The effects of roughness on the characteristics of 3T3 cells were opposite to the result for XB-2 cells. E1 proliferative ability did not differ with any statistical significance. These results suggest that a rougher surface which provided calcium and phosphate ions have the ability to enhance the proliferation of osteoblast and the inhibition of fibroblast growth that enhance implant success ratios.

## 1. Introduction

Successful dental implant treatment depends on three components: bone, connective tissue, and epithelium. Each plays an important function; for example, connective tissue cannot anchor the implant surface having mechanical attachments as bone does because inadequate peri-implant epithelium function can lead to the creation of deep pockets and invasion of bacteria [1–3]. In addition, the fundamental esthetic outcomes associated with dental implant treatments are related to the healing characteristics of both the epithelium and connective tissue [4]. Different surface designs of materials can also cause varying levels of peri-implant hard and soft tissues [4, 5]. Accordingly, the chemical composition and physical properties of the implant's surface can affect peri-implant tissues behavior [6, 7].

Many methods are used to create a rough implant surface. To create a three dimensional roughness on the implant surface for improving bone anchorage, differential ways such as titanium plasma spraying, grit or sandblasting, acid-etching, and anodization were used [7–9]. Among these, a most popular way of the sandblasting and acid etching (SLA) have been reported. The SLA treatment showed the properties of earlier osseointegration and decreased bone loss than other surface treatment ways [10]. However, harmful metal ion dissolution and particles such as aluminum remain in the processes by SLA surface treating and lead to local or systemic toxic effects [11–13].

**Figure 1:**
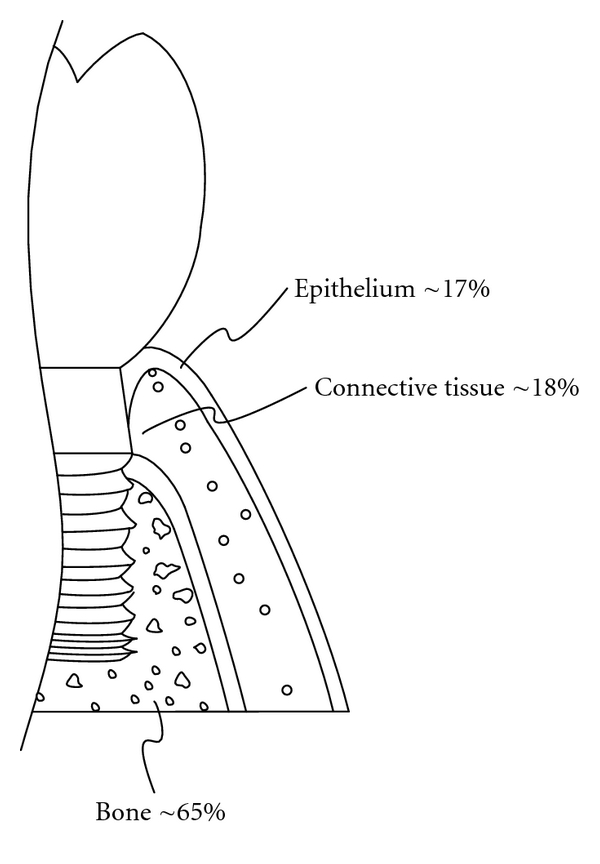
A diagrammatic illustration showing the relationship of the interfacial parts between periodontal tissues and an implant (length: 5.7 mm). The relationship between the bone of the alveolar process, the gingival connective tissue and junctional epithelium are shown, at the level where the implants and tissues contact.

Peri-implant soft tissues play an important role, as they might encompass over one-third of the height area of short implants (Figure 1). After implantation, two distinct responses may occur on the implant surface. The bone tissue can contact the implant surface with a proper biological width, signaling a successful treatment with complete osseointegration. Another response is fibrous encapsulation involving the soft tissue covering the entire implant surface. These responses involve the three aforementioned cell types, which have distinct growth patterns and varying abilities of adhesion to the implant surface and the early stage attaching and repopulating abilities of cells is a common technique for evaluating initial stability of the implant [14–16].

Because implant surface treatment is usually only concerned with integration of bone tissues and neglects soft tissues, the surfaces contacting the implant and surrounding tissues deviates from the originally intended design leading to implantation failure. For this reason, current techniques are insufficient to achieve controlled success. Hence, the purpose of this study is to find the cells relationship to develop a proper tissue architecture on the pure Ti implant surfaces that is almost identical to the patient's original after tooth extraction and implantation. The process was to treat the different roughen surfaces, and then samples were cultured with bone cells, fibroblasts, and epidermal cells to evaluate the differential early stage cell attaching and repopulating abilities.

## 2. Materials and Methods

### 2.1. Materials—Surface Treatment

Commercially pure, grade II titanium (cp Ti) samples (Buehler Ltd, USA) measuring 6 × 5 × 1 mm in respective length, width, and thickness of constant surface areas of 30 mm^2^ within 2.5% standard deviation (SD) were used. The samples were embedded into an epoxy resin to be polished by sandpaper of decreasing grain sizes: 400, 1200 and 2000. Then the samples were washed following with ethanol, acetone, and distilled water using ultrasonic oscillation for each 5 min. The Ti metal surface, a control group, controlled an Ra value of 0.12 micrometer (*μ*m) within 5.0% SD.

Testing groups, in which the surfaces of the control group were sandblasted for 10, 20, and 30 sec with aluminum (Al_2_O_3_) particles (mean size 54.5 ± 32.1 *μ*m). The sandblasting used an air compressor with 7 kg/m^2^ of powder blasted over a 0.5 mm distance. After sandblasting, the samples were acid-etched for 30 sec. An etching solution of the HCl (37%, Panreac, Barcelona, Spain) to H_2_SO_4_ (95–98%, Panreac, Barcelona, Spain) to H_2_O volume ratio of 1 : 1 : 1 was used. Testing groups were after different blasting times and following acid-etching processes (SLA) for constant time 30 sec were presented as the symbols of 10/30, 30/30, and 60/30 groups (*n* = 10). To clarify the ion effect of 10/30 testing group, a comparative group receiving secondary grit-blasting using TTCP particles (10 sec), which was prepared in-house and had a mean particle size of 10.1 ± 0.7 *μ*m [17], was present as a SLA 10/30/TTCP group. The 10/30/TTCP group was dry-heat sterilized at 160°C for 2 h and the other samples were sterilized in an autoclave.

### 2.2. Surface Characterizations

The average surface roughness was measured using a roughness tester (SJ-301 Mitutoyo Ltd, Japan) and presented in Ra. Topographies and the anchored/residual particles were analyzed by a scanning electron microscope (SEM, Hitachi S-3000N, Japan) equipped with an energy dispersive X-ray spectrometer (EDS, Horiba EX220, Japan).

### 2.3. Cell Abilities and Morphologies

Three cell lines of the bone (MC3T3-E1, abbreviated E1), fibroblast (NIH 3T3, abbreviated 3T3), and epidermal (XB-2) cells were provided by the National Institute of Health (NIH) in Taiwan. E1 was derived from newborn mouse calvaria and were cultured in a 10 mL of *alfa*-modified Eagle's medium (MEM) containing 10% fetal bovine serum (FBS) (Biological Industries, Haemek, Israel), and 1% penicillin (100 units/mL)/streptomycin (100 *μ*g/mL) (Gibco, Invitrogen Taiwan Ltd., MD). 3T3 was derived from newborn mouse fibroblasts and cultured in Dulbecco's modified Eagle's medium (DMEM) (Gibco, Invitrogen Taiwan Ltd., MD) containing 10% bovine serum (BS) (Biological Industries, Haemek, Israel). XB-2 was derived from newborn mouse keratinized cells and cultured as in the study [18]. XB-2 cells were grown in the presence of 3T3 cells, which were briefly cultured on a 0.1% gelatin-coated plate (G932-500G, Sigma Co., St. Louis, USA) before culturing in DMEM containing mitomycin C (10 *μ*g/mL) for 2.5–3 h. Subsequently, XB-2 cells were cultured in DMEM containing 20% FBS. All cells were cultured in a humidified atmosphere in a 5% CO_2_ incubator. The culture medium was replaced every 2-3 days. After the cultured cells were harvested, cells were counted and seeded on the prepared surfaces at 1 × 10^5^cells/sample.

An XTT Cell Viability Assay Kit provided a simple method to count live cells using an absorbance reader. The cells' adhesive and repopulative abilities were measured at two early stage time points of 1 h and 24 h. After the cultured time, the cells on the samples' surface were washed with phosphate-buffered saline (PBS) and transferred to a 200 *μ*L culture medium with a 100 *μ*L XTT kit and were incubated for another 4 h. The reaction medium was then measured spectrophotometrically at 490 nm using an ELISA microplate reader UVM-340 (ASYS Hitech GmbH, Eugendorf, Austria). Finally, the cell numbers were determined from a plot of absorbance (OD values) versus the respective E1, 3T3, and XB-2 cells after adjustment via XTT assays. Each experiment was performed five times (*n* = 5).

After being cultured, the samples were washed and fixed with a mixture of 2% paraformaldehyde and 2.5% glutaraldehyde for 2 h. After dehydration in a graded series of ethanol, the samples were treated with iso-amyl acetate and dried using a critical point dryer. The specimens were sputter-coated with gold and the cell morphology was observed using SEM. To compensate for the ion effects in the medium, TTCP was extracted at a ratio of 1 g TTCP to 10 mL culture medium. The three cells were cultured in the extraction and on the selected 10/30/TTCP surfaces for 24 h. The statistical analysis was performed using JMP 6.0 software (SAS Institute Inc., Cary, NC, USA) with statistical significance set at *P* < 0.05.

## 3. Results

### 3.1. Topographies and Elements Mapping

**Figure 2:**
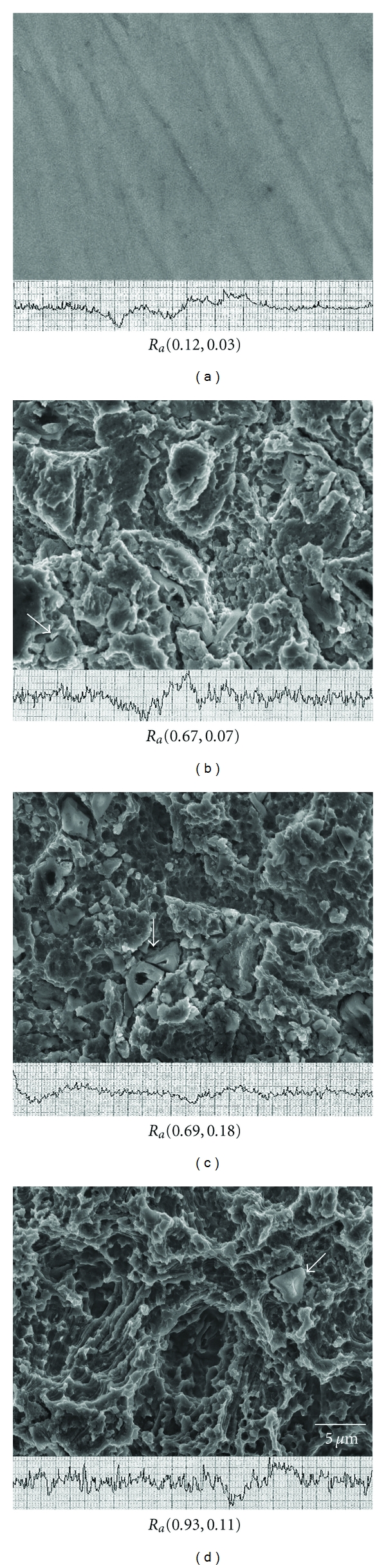
The different topographies of cp Ti surfaces and their respective levels of roughness after blasting/etching for various times (sec). (a) Control and (b) 10/30, (c) 30/30, (d) 60/30 test groups. Ra: average roughness (mean, SD), units: micrometers. Arrowhead: surface-trapped alumina.

**Figure 3:**
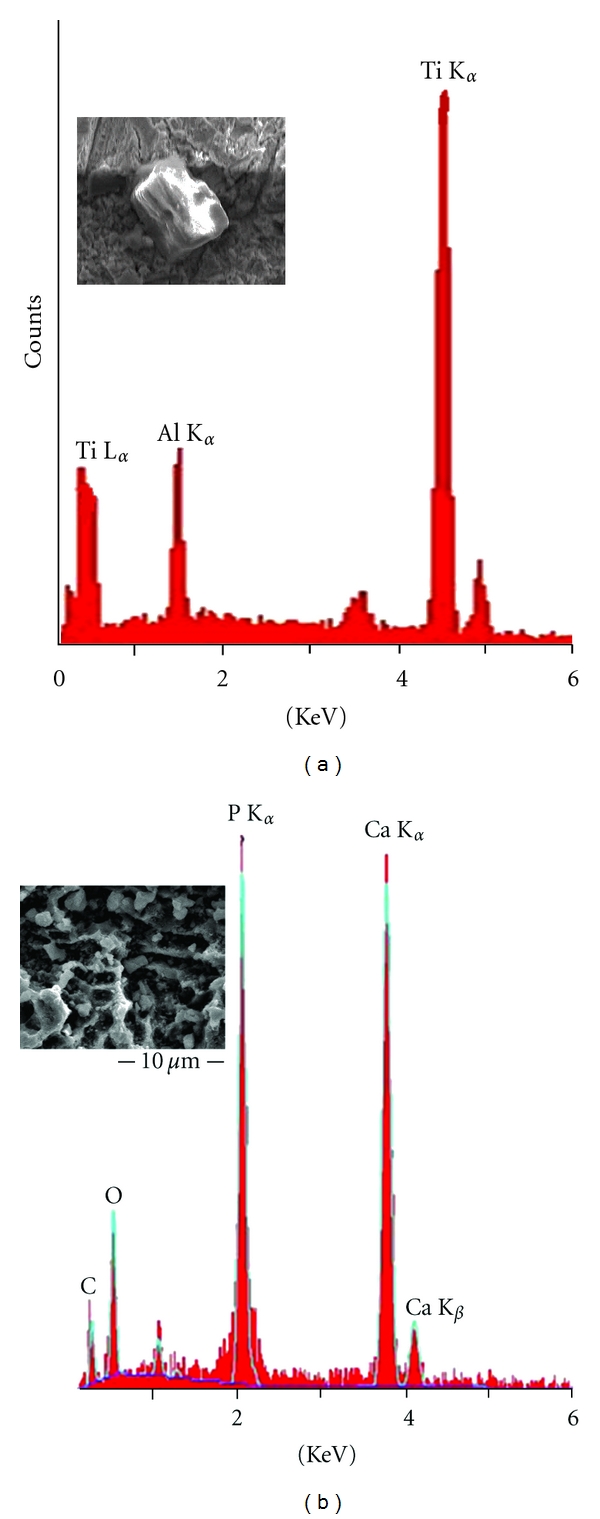
(a) Results of spectrum showing the surfaces having alumina particles in the test groups after 10/30 sec of blasting/etching. (b) EDS showing the surfaces having high levels of Ca and P atoms without alumina through secondary blasting of TTCP particles.

The flattest surface was observed in the control group and the etching effects on the Ti surfaces became more significant as the blasting time was increased in Figures 2(b)–2(d). The SLA 60/30 group obviously increases the roughness more than other groups. Al_2_O_3_ particles are still captured or anchored on the surface even after 5 min ultrasonic cleaning accompanied by 30 sec etching. However, Al_2_O_3_ particles were not found after the secondary grit-blasting using TTCP particles (Figure 3(b)), where only calcium and phosphorus elements were found and reduce the Ra values in the groups from SLA test group to SLA 10, 30, and 60/30/TTCP (Ra 0.62 ± 0.07, 0.63 ± 0.09, and 0.71 ± 0.08).

### 3.2. Early Stage Cell Properties

Cells were cultured for 1 h and 24 h each to determine their adhesive and initial proliferative abilities. Statistical analysis of all the groups was shown in the Table 1.

**Figure 4:**
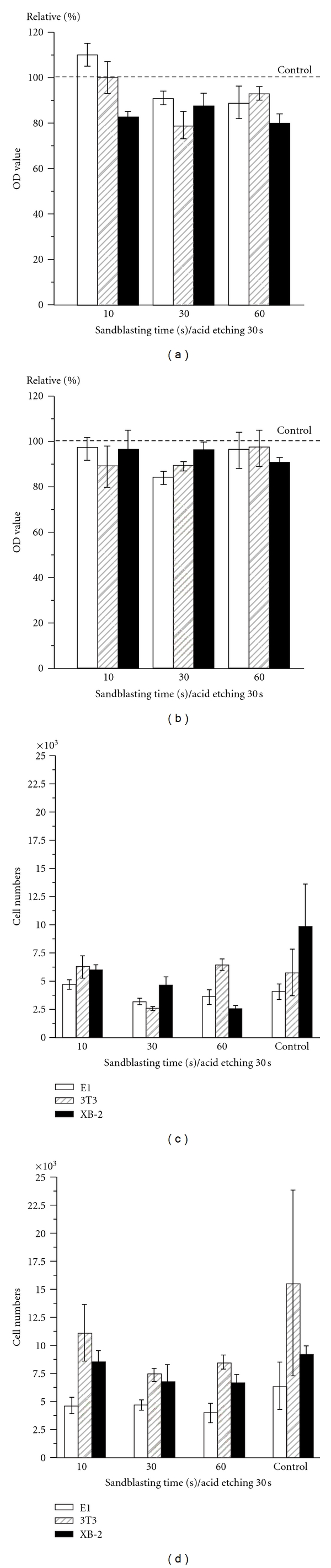
Different cells cultured on a variety of topographies: OD values after (a) 1 h of adhesion and (b) 24 h of proliferation were compared with the control group and the cell numbers were counted after 1 h (c) and 24 h (d).

Accordingly to the OD values (Figures 4(a) and 4(b)), higher Ra values would reduce the OD values of the E1 and 3T3 cells, however, XB-2 cells were unaffected in all measurements. When the cell numbers were aligned and counted after 1 h (Figure 4(c)), the XB-2 cells on the flattest surface was shown to be four times larger than on the rough SLA 60/30 surface. After 24 h, XB-2, and 3T3 cells behaved differently depending on the Ra values. As such, the behavior of 3T3 cells was not obviously different after 1 h and 24 h of SLA 60/30. The epidermal cells proliferate faster on the rough surface and the fibroblasts displayed a contrary proliferation tendency on a surface with 60/30 SLA treatment, the number of XB-2 cells obtained after 1 h increased 2.6 times after 24 h (Table 1).

### 3.3. Morphologies of Cells

**Figure 5:**
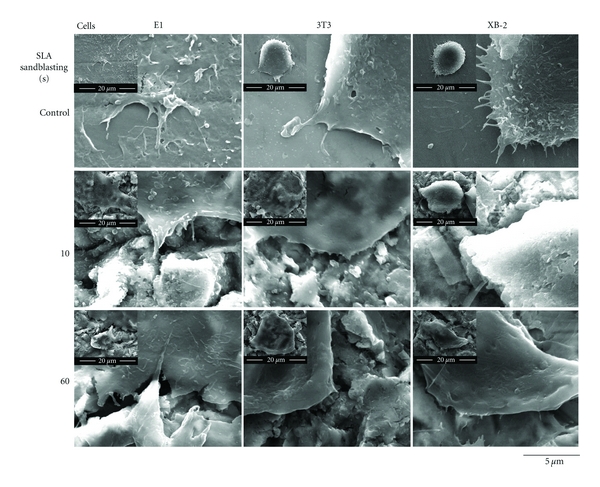
SEM images showing the proliferative morphologies of the various cells growing on areas of varying roughness in the control and test groups after 10, 30, and 60/30 sec of blasting and etching after 24 h.

XB-2 and 3T3 cells displayed a round morphology and the filopodia of E1 demonstrated a spider shape originally. The XB-2 and 3T3 cells in the control group were spindle-shaped and especial the XB-2 cells, proliferated on the largest Ra samples, had an entirely different morphology (Figure 5). The filopodia were extended and evenly distributed over the surface. This phenomenon of altered epithelial morphology is indicating that XB-2 cells have a better growth rate on the rough surface [19].

### 3.4. Ions Effect

**Figure 6:**
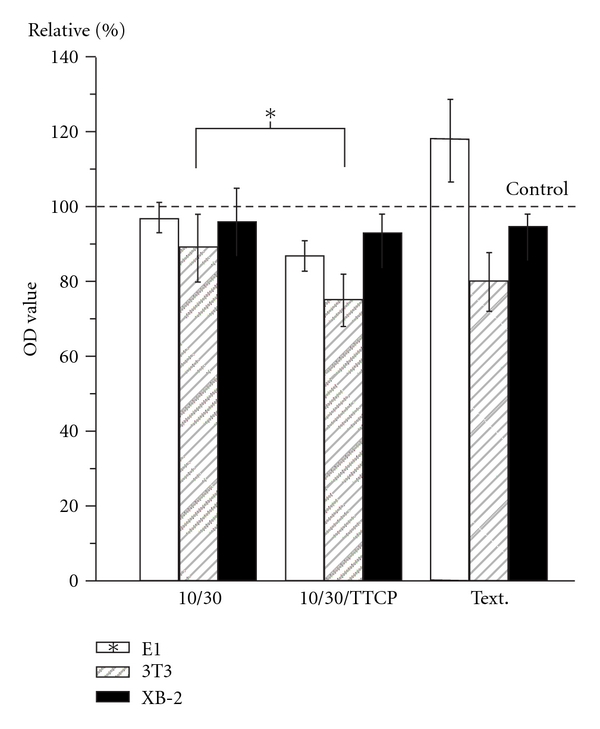
A diagram indicating the OD values after 24 h cell proliferations of the test groups: SLA 10/30, 10/30/TTCP and TTCP extraction cells culture medium (Text.) (*: a statistically significant difference in the group comparisons, *P* < 0.05).

Proliferative patterns of XB-2 and 3T3 cells were no different among SLA 10/30, SLA 10/30/TTCP, and TTCP-extracted medium groups and the conditions of E1 cells deteriorated with anchored SLA 10/30/TTCP group (*P* < 0.05) (Figure 6). However, TTCP-extracted culture medium is basically aid for the E1 early stage proliferation at 24 h cultured. Hence, in the early stages of cell repopulation, surface conditions are clearly more important for bone cells than the effects of ions.

## 4. Discussion

To view the SLA procedures, Al_2_O_3_ is widely used as sandblasting particles for surface cleaning or developing roughness. These particles, which were entrapped in the Ti surface, were difficult to remove through a popular used single acid-etching process. Moreover, this has been proved that can cause poor osseointegration, and a high density of Al ions on the Ti alloy may be related to Alzheimer's disease [20–22]. Fortunately these particles can be replaced by a secondary sandblasting technique, which has a smaller particle size distribution than Al_2_O_3_.

After placement of the dental implant, the complications of infection and fibrous encapsulation may occur during the healing process. The success of dental implants cannot merely be defined by the efficacy of osseointegration between the bone and the implant. Rather, a proper biological interaction to obtain healthy gingiva is essential as shown in Figure 1. Among both of these contact parts between the tissues and the implant, epithelium linking would lead to the absence of inflammation [14]. The integration of soft tissue provides a beneficial strategy where the epithelium linking is enhanced while the contact part of the gingival connective tissues is suppressed.

Unlike the case with their early stage adhesive and proliferative behavior, the XB-2 and 3T3 cells acted differently than each other in respect to their reaction to surface roughness. Based on the analysis in Figures 4(c) and 4(d), 3T3 cells cultured on the smooth surface of the control group after 1 h were not significantly different from the largest roughness SLA 60/30 testing group (Table 1). However, in the control group, after 24 h cultured, the 3T3 cells went largely beyond testing groups with rough surfaces and were a 2.7-fold increase in cell numbers at 1 h culturing. Contrary to the results obtained with the 3T3 cells, the XB-2 cells had a statistical significance in roughness after 1 h of culturing; the control group displayed the best cell adhesive ability. However, after the XB-2 cells were allowed to proliferate at early stage for 24 h, there were no significant differences between the control and test groups (*P* > 0.05). This early stage result indicates that epithelial cells cultured on roughened surfaces have better proliferative abilities than those cultured on smooth surfaces. The proliferative rate of XB-2 cells increased 2.6-fold from 1 h to 24 h of culturing for the SLA 60/30 testing, though the control group showed no specific statistical change. XB-2 cell's qualities in the control group after 1 h culturing were better than those of the roughened surfaces, the proliferative properties after 24 h culturing were reversed.

 Early stage cell abilities such as adhesion and proliferation to the substrates can vary according to surface topography, which in turn influences cytoskeletal components [19, 23]. Sandblasting was thought to induce stress on the surface, whereas acid etching was thought to release the resulting residual stresses. Several related experiments [16, 24–26] reported that surface conditions can affect different types of cell morphology, and this is referred to as cell-specific discrepancies [27]. Surface characteristics have been shown to regulate how the different cells reach clinically appropriate proportions with respect to the implant. For example, one of the main challenges in implant treatment lies in achieving an esthetic appearance, involving a physiological outcome [28–30]. For soft-tissue integration involving epithelium cells adhesion and proliferation, roughened topographies should be recommended, as they can increase linking and inhibit the risk of fibrous capsulation by connective tissues.

 The ion effects are demonstrated in Figure 6. The early stage cell proliferative ability in the extracted cultured medium was significantly increased (*P* < 0.05) in E1 cells but decreased in 3T3 cells. However, when the ionic effect was combined with the effect of topography in the comparative SLA 10/30/TTCP group, the proliferative ability of E1 cells was shown to be significant lower (*P* < 0.05) than in the SLA 10/30 group. The calcium and phosphate ions became incorporated into the apatite that formed in an intimate association with the organic component, leading to bone formation [31, 32]. Figure 6 showed the promoted E1 bone cell growth was largely due to the topography rather than the ions. The ion effect played a less important role than the roughness with respect to E1 cell proliferation. The existence of calcium phosphates in a thin film coating can play a mediatory role between implants and natural bone tissues, but such properties did not lead to clinical success [33, 34]. Rough surfaces enhanced the ability to act as essential factors for bone cell adhesion and proliferation. The contacting surfaces between implants and the soft tissues should be roughened and ions releasing from TTCP hydrolysis should also be commented. This study clearly confirms the hypothesis that roughness and ion effects would impact the initial implant stability by the early stage cell interactions. The results had demonstrated that TTCP as the ions releasing medium could be a potential application in bone regeneration and prevention of the fibrous encapsulation of implants.

## 5. Conclusions

Techniques necessary to harmonize the early stage adhesion and proliferation of osteoblasts and epithelial cells on the implant are important. The optimal implant can be designed with a smooth surface in the top area of implant, which is closer to the gingival surface, to promote rapid epithelial cell adhesion that could lead to prevent inflammation after implantation. A rougher surface anchored with TTCP can replace Al_2_O_3_ particles in the sandblasting process and provide soluble ions to enhance the early stage proliferation of osteoblast cells. Such results suggest that an active surface can be prepared to achieve appropriate implant biological widths. In summary, we emphasize a concept whereby an implant actively regulates cells rather than undergoing a passive healing process, at the same time eliminating the dangers of fibrous encapsulation at an early implant stage.

## Figures and Tables

**Table 1a:** (a)

Statistical analysis*	Variations	Time of sandblasting (sec)							
		0^b^		10		30		60	
Groups	Cells	*P* value	Group comparisons^a^	*P* value	Group comparisons	*P* value	Group comparisons	*P* value	Group comparisons
1 h cell adhesion	3T3/E1/XB-2	0.0161	XB-2 > E1	0.0077	3T3 > E1 XB-2 > E1	0.0013	XB-2 > E1 XB-2 > 3T3	<0.0001	3T3 > E1 3T3 > XB-2 XB-2 > E1
24 h cell proliferation	3T3/E1/XB-2	0.0705	none	0.0026	3T3 > E1 XB-2 > E1	0.0231	XB-2 > E1	<0.0001	3T3 > E1 3T3 > XB-2 XB-2 > E1

**Table 1b:** (b)

Statistical analysis*	Variations	E1 bone cell		3T3 fibroblast cell		XB-2 epidermal cell	

Groups	0 sec^b^ and through 30 sec etching after 10, 30, and 60 sec blasting	*P* value	Group comparisons^a^	*P* value	Group comparisons^a^	*P* value	Group comparisons^a^
1 h cell adhesion	0/10/30/60	0.0025	30 > 0, 10, 60	0.0026	10 > 30 60 > 10	0.0005	0 > 10, 30, 60 10 > 60
24 h cell proliferation	0/10/30/60	0.0510	none	0.0791	none	0.2788	none

*Groups significantly differ at *P* < 0.05; ^a^“none” indicates the group comparisons are not significantly different at *P* > 0.05; ^b^“0 sec” indicates the control group without the blasting/etching treatment.
